# Sustainability of mHealth Effects on Cardiometabolic Risk Factors: Five-Year Results of a Randomized Clinical Trial

**DOI:** 10.2196/14595

**Published:** 2020-04-21

**Authors:** Antonio Bernabe-Ortiz, Julia Pauschardt, Francisco Diez-Canseco, J Jaime Miranda

**Affiliations:** 1 CRONICAS Center of Excellence in Chronic Diseases Universidad Peruana Cayetano Heredia Lima Peru; 2 School of Medicine Universidad Peruana Cayetano Heredia Lima Peru

**Keywords:** mHealth, low- and middle-income countries, blood pressure, body weight

## Abstract

**Background:**

The long-term effects of mobile health (mHealth) interventions have not been documented, especially in resource-constrained settings.

**Objective:**

This study aimed to assess the effects of a 1-year mHealth intervention on blood pressure levels and body weight in low-resource urban settings in Peru, 4 years after the completion of the original study.

**Methods:**

Four years after the original Grupo de Investigación en Salud Móvil en America Latina (GISMAL) study, we attempted to contact the 212 individuals originally enrolled in the study in Peru. The primary outcomes were systolic and diastolic blood pressure levels and hypertension incidence. Secondary outcome measures were body weight, BMI, and self-reported target behaviors. The study personnel collecting the data were masked to the group assignment. Linear mixed models were used to evaluate the effects of the intervention on primary and secondary outcomes in an intention-to-treat analysis.

**Results:**

Data from 164 (77.4%) of the 212 originally enrolled participants were available and analyzed (80 in the intervention group and 84 in the control group). The intervention did not result in changes in systolic (–2.54 mm Hg, 95% CI –8.23 to 3.15) or diastolic (3.41 mm Hg, 95% CI –0.75 to 7.57) blood pressure compared with the control group. The intervention reduced the risk of developing hypertension, but the result was not significant (risk ratio (RR) 0.76, 95% CI 0.45-1.28). However, those who received the intervention had lower body weight (–5.42 kg, 95% CI –10.4 to –0.48) and BMI (–2.56 kg/m2, 95% CI –4.46 to –0.66). In addition, compared to the control participants, those who received ≥50% of the scheduled calls during the intervention had greater reductions in body weight (–6.23 kg, 95% CI –11.47 to –0.99) and BMI (–2.81 kg/m2, 95% CI –4.77 to –0.85).

**Conclusions:**

An mHealth intervention comprising motivational interview calls and SMS text messaging appears to have effects on health 4 years after intervention completion. Although there were no effects on blood pressure levels, important reductions in body weight and BMI were seen 5 years after randomization. Thus, mHealth appears to be a promising preventive strategy for noncommunicable diseases in resource-constrained settings.

**Trial Registration:**

Clinicaltrials.gov NCT01295216; https://clinicaltrials.gov/ct2/show/NCT01295216

## Introduction

Hypertension is the most important risk factor for stroke and premature cardiovascular disease (CVD) [1,2]. Prehypertension is defined as a systolic blood pressure (SBP) of 120-139 mm Hg or a diastolic blood pressure (DBP) of 80-89 mm Hg [3]; however, the risks of coronary artery disease and stroke rise progressively as blood pressure increases above 115/75 mm Hg [4]. As a result, interventions focused on individuals with prehypertension may be of interest to address the burden of hypertension.

Worldwide, the number of individuals who own a mobile phone is increasing. Mobile health (mHealth), an ever-expanding concept, uses this growing technology in a wide range of health care applications [5]. Interventions using mHealth have the potential to shorten gaps to reach underserved populations [6], providing a more flexible platform for improving patient self-care. mHealth technology has been applied successfully to meet the treatment of infectious chronic diseases such as tuberculosis (treatment adherence, prevention, and education) [7] and HIV/AIDS (uptake of sexual health services and information) [8], and some mHealth interventions have been used to promote changes toward healthier lifestyles, thereby improving health outcomes [9-11]. However, the number of studies assessing the impact of mHealth on specific cardiovascular outcomes is more limited in low-income and middle-income settings [6,12], and although the success of these interventions is evident, the long-term impact of interventions involving mHealth technology is not.

Some interventions have been proved to be effective to change lifestyle behaviors. A systematic review found that interventions based on the Transtheoretical Model can reduce fat consumption, increase the consumption of fruit and vegetables, and increase physical activity depending upon the progression through the stages of change [13]. Similarly, a recent systematic review reported that telephone-based interventions that incorporate motivational interviewing are promising for weight loss [14]. The GISMAL (Grupo de Investigación en Salud Móvil en America Latina, in Spanish) study was a 1-year randomized controlled trial conducted in three Latin American countries (Argentina, Guatemala, and Peru) using the Transtheoretical Model and motivational interviewing.

The aim of the GISMAL study was to assess whether an mHealth intervention would improve the cardiometabolic profile (ie, reduce blood pressure levels and body weight) among individuals with prehypertension [15,16]. Although the intervention did not reduce blood pressure levels, it was associated with a reduction of body weight and improvement in some dietary habits, especially in Peru. One year may not be a sufficient period to observe changes in blood pressure levels due to a behavioral intervention or to assess whether the effects of the intervention can continue to provide benefit after it is stopped; therefore, we aimed to evaluate the long-term effects (ie, 4 years after completion of the original study) of the GISMAL mHealth intervention on blood pressure and body weight in participants recruited in Peru.

## Methods

### Original Intervention and Settings

The GISMAL study (NCT01295216) was performed in 2012. It was a multicenter, parallel-group, randomized controlled trial that was stratified by sex and age (30-44 years and 45-60 years). Details about the intervention have been published elsewhere [15]. In brief, randomization was stratified by country using minimization by sex and age group. The intervention lasted 12 months, followed a standardized protocol, which was implemented by trained nutritionists and comprised monthly phone calls in which the nutritionists used motivational interview techniques. Participants were contacted through their personal mobile phones, and the conversations focused on reduction of dietary sodium intake, reduction of high-fat and high-sugar food intake, increase in fruit and vegetable consumption, and promotion of physical activity. In addition, SMS text messages were sent weekly to participants to reinforce the calls [17]. The same nutritionist entered the information obtained during each call into a web-based platform to customize the weekly text messages delivered to participants in the following month. Several text messages were developed and culturally adapted to each country to guarantee understanding, adequacy of the message wording, and tone, as previously described [18].

Eligibility criteria included men and women aged 30-60 years who owned mobile phones and with SBP and DBP in the prehypertension range (between 120 and 139 mm Hg and between 80 and 89 mm Hg, respectively). People with an earlier diagnosis of diabetes or hypertension, illiterate individuals, and pregnant women were excluded.

The total sample of the original intervention was 636 people from Argentina, Guatemala, and Peru; however, the sample size of the trial was calculated separately for each country. Thus, researchers from Peru enrolled 212 individuals to ensure a change in systolic blood pressure levels of 5 mm Hg. Subjects from two different sites (Pampas de San Juan de Miraflores and Hospital Nacional Cayetano Heredia) were recruited; 107 were allocated to the intervention group and 105, to the control group. The outcomes of interest were blood pressure, body weight, diet quality, and physical activity; these were evaluated at baseline (randomization) and at 6 and 12 months later.

### Follow-up Assessment

Between August and December 2017, on average, 5 years after randomization (ie, 4 years after completion of the intervention), participants enrolled in Peruvian sites were contacted to determine the long-term effects of the intervention (ie, whether the effects of the mHealth intervention were maintained over time and affected blood pressure levels as originally planned). In the time between the 1-year and 5-year assessments, the research team had no contact with the participants. As per the original study [15], where participants had moved or had changed their telephone number, family members or friends reported as next of kin in the original study were contacted to find the participants.

### Primary and Secondary Outcomes

As in the original study, in this new assessment, the primary outcomes were SBP and DBP, both measured in millimeters of mercury; in addition, hypertension incidence was included as a primary outcome. The secondary outcomes were the same as in the original study, including body weight (in kilograms), BMI (kg/square meter), physical activity (in metabolic equivalents [METs] per minute of a task per week), and diet patterns (daily intake of fruits and vegetables, high-sodium food, and of high-fat and high-sugar foods).

SBP and DBP were measured using an automatic monitor (HEM-742 INT, Omron) as in the original study. Measurements were taken in triplicate; after a 5-minute resting period, the first blood pressure measurement was taken, and the time between subsequent measurements was at least 1 minute. The average of the second and third blood pressure measurements was used for the analyses. All measurements were taken with the participant in a seated position using the same arm where the original measurement was taken.

Body weight was measured three times, following standardized techniques. We used the same digital scales (Seca 803/Omron SC-100) used in the original study and calibrated them to an accuracy of 100 g. BMI was calculated by dividing weight (kg) by height (m) squared. Primary and secondary outcomes were evaluated as numerical variables; however, hypertension incidence was evaluated by taking into account the new cases of hypertension detected since randomization.

Changes in physical activity were evaluated using the METs obtained from the short version of the International Physical Activity Questionnaire [19]. Moreover, diet patterns were evaluated using the same food frequency questionnaire used in the original study; the questions focused on the consumption of foods with high contents of sodium, simple sugars, trans fats, and saturated fats, as well as on the consumption of fruits and vegetables [20].

Other important variables were considered, including sociodemographic data (age, sex, marital status, household income, years of education, employment status, and health insurance coverage) as well as self-reported lifestyle behaviors (smoking status, alcohol intake, physical activity, and daily dietary intake). In addition, stages of change (precontemplation, contemplation, preparation, action, and maintenance) according to the Transtheoretical Model [21] were described (physical activity, intake of servings of 5 fruits and vegetables, food with harmful fats, high-sugar food and beverages, high-sodium processed foods, salt added at the table, and salt added for cooking).

### Statistical Analysis

Data were analyzed using STATA 13 software (Stata Corp). The intention-to-treat principle was used to compare the intervention and control arms. Means and standard deviations for numerical continuous variables and proportions and frequencies for categorical variables were used to describe the study population enrolled in Peru at baseline. 

For the incidence analysis, we excluded patients whose hypertension status could not be confirmed, including those who were lost to follow-up and who had died. Person-years of follow-up were calculated by summing the follow-up times for the remaining participants. For participants with a new diagnosis of hypertension, only half of the time between the last assessment and the previous assessment was used in this sum since the actual date of diagnosis was unknown. Incidence rates and their 95% confidence intervals were then estimated. Poisson regression models with log link functions and robust standard errors to account for cluster effects were calculated by reporting the risk ratio (RR) and 95% confidence intervals.

Differences in primary and secondary outcomes were assessed using linear mixed models, including two levels (assessments as level 1 units and subjects as level 2 clusters). As in the original study, the a priori defined model included the interaction between the intervention and the time of follow-up as categorical variables (baseline, 6 months, 12 months, and 5 years), adjusting by sex and age to reverse the stratification during randomization. The regression model results focus on the 5-year assessment and are presented as coefficients with their respective 95% CI. A dose-response analysis was also conducted using appropriate statistical techniques but was categorized in two groups instead of the three groups in the original study: participants who received <6 (50%) and ≥6 (50%) of the scheduled motivational interviewing calls.

### Ethical Aspects

As we only contacted participants from the Peruvian sites, this new assessment was approved by the Ethics Committee of the Universidad Peruana Cayetano Heredia, Lima, Peru. To ensure the autonomy of the participants, written informed consent was obtained before we assessed the participants. The data collected were kept confidential and accessed only by the researchers who performed the study.

## Results

### Principal Findings

**Figure 1:**
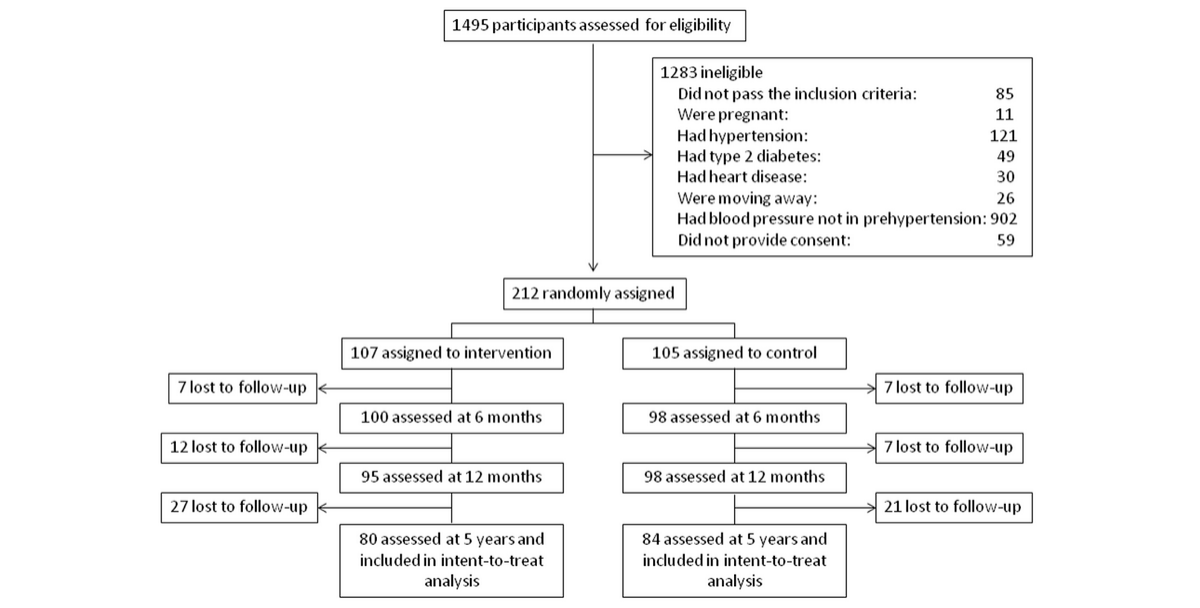
Trial profile in Peru.

In Peru, a total of 1495 participants were assessed for eligibility; finally, 212 were randomly assigned to the intervention or control arm. After the first year of the study, 193 participants were retained in the study (n=95, 49.2% in the intervention group and n=98, 50.8% in the control group); 5 years after randomization, 1 (0.5%) participant had died, and 47 (22.2%) were lost to follow-up. Therefore, data from 164 individuals were analyzed (n=80, 48.8% in the intervention group and n=84, 51.2% in the control group; Figure 1).

According to the baseline comparison, the randomization works relatively well, as the distributions of the characteristics of the study population were similar in the intervention and control groups (Multimedia Appendix 1).

### Primary and Secondary Outcomes

In intention-to-treat analysis, the intervention did not result in changes in SBP (–2.54 mm Hg, 95% CI –8.23 to 3.15) or DBP (3.41 mm Hg, 95% CI –0.75 to 7.57) compared to controls. Among secondary outcomes, those who received the intervention had lower body weight (–5.42 kg, 95% CI –10.4 to –0.48) and BMI (–2.56 kg/m^2^, 95% CI: –4.46 to –0.66) compared to the control group. However, there was no evidence of effects of the intervention on physical activity levels, intake of fruits and vegetables, or intake of high-sodium food, but there were small increases in the intake of high-fat and high-sugar foods (Table 1).

The intervention reduced the risk of developing hypertension after 5 years of follow-up (RR 0.76, 95% CI 0.45-1.28); however, the estimates were not significant (Table 2).

**Table 1:** Primary and secondary outcomes at 5 years of follow-up: results from mixed linear models.

Outcome		Intervention group (n=80)	Control group (n=84)	Mean difference^b^ β (95% CI)	*P* value	
**Primary**						
	Systolic blood pressure	124.0 (12.1)	126.6 (12.5)	–2.54 (–8.23 to 3.15)	.38	
	Diastolic blood pressure	74.4 (8.8)	74.2 (9.0)	3.41 (–0.75 to 7.57)	.11	
**Secondary**						
	Body weight (kg)	78.0 (15.1)	79.8 (15.7)	–5.42 (–10.4 to –0.48)	.03	
	BMI (kg/m^2^)	31.4 (5.2)	32.8 (5.9)	–2.56 (–4.46 to –0.66)	.008	
	Physical activity (METs^a^/min per week)	1077 (1641.6)	911 (1274.6)	–61.12 (–745.94 to 623.70)	.86	
	Daily intake of fruits and vegetables^b^	1.62 (0.95)	1.65 (0.94)	0.05 (–0.47 to 0.56)	.86	
	Daily intake of high-sodium food^b^	0.56 (0.60)	0.82 (0.69)	–0.22 (–0.55 to 0.11)	.20	
	Daily intake of high-fat and high-sugar foods^b^	3.72 (1.69)	3.93 (1.73)	0.98 (0.14 to 1.82)	.02	

^a^METs: metabolic equivalents.

^b^Mean differences were estimated using linear mixed models using information from the baseline and 6-month, 12-month, and 5-year follow-ups and were controlled by sex and age.

**Table 2:** Incidence of hypertension by intervention group and dose-response to intervention 5 years after randomization.

		New cases of hypertension during follow-up			Regression model^a^ RR (95% CI)		*P* value
		No (n=142)	Yes (n=50)				
**Intention-to-treat analysis**							
	Control	69 (70.4%)	29 (29.6%)	1 (Reference)		—	
	Intervention	73 (77.7%)	21 (22.3%)	0.76 (0.45-1.28)		.30	
**Dose-response analysis**							
	Control	69 (70.4%)	29 (29.6%)	1 (Reference)		—	
	<50% (<6 calls)	20 (66.7%)	10 (33.3%)	1.33 (0.64-2.75)		.44	
	≥50% (≥6 calls)	53 (82.8%)	11 (17.2%)	0.55 (0.29-1.04)		.07	

^a^Models were adjusted by age and sex, as randomization was stratified for these variables.

### Dose-Response Analysis

No changes in SBP (–4.05 mm Hg, 95% CI: –10.11 to 2.02) or DBP (1.48 mm Hg, 95% CI: –2.84 to 5.79; Table 3) were observed among participants receiving ≥50% of motivational calls. On the other hand, those receiving ≥50% of scheduled calls during the intervention had a 45% reduction (RR 0.55, 95% CI: 0.29-1.04) in the risk of developing hypertension 5 years after randomization; however, the estimates were not significant (Table 2).

Among secondary outcomes, participants in the intervention group who received ≥50% of calls had greater reductions in body weight (–6.23 kg, 95% CI: –11.47 to –0.99) and BMI (–2.81 kg/m^2^, 95% CI: -4.77; -0.85); however, there were no changes in the other secondary outcomes (Table 3).

**Table 3:** Dose response to intervention in primary and secondary outcomes at 5-year follow-up.

		Mean difference (<50%/<6 calls)^b^ (n=26)		Mean difference (≥50%/≥6 calls)^b^ (n=54)	
		β (95% CI)	*P* value	β (95% CI)	*P* value
**Primary outcomes**					
	Systolic blood pressure	0.42 (–5.89 to 6.72)	.90	–4.05 (–10.11 to 2.02)	.19
	Diastolic blood pressure	7.13 (2.31 to 11.95)	.004	1.48 (–2.84 to 5.79)	.50
**Secondary outcomes**					
	Body weight (kg)	–3.57 (–10.47 to 3.34)	.31	–6.23 (–11.47 to –0.99)	.02
	BMI (kg/m^2^)	–1.79 (–4.45 to 0.87)	.19	–2.81 (-4.77; -0.85)	.005
	Physical activity (METs^a^/min per week)	–163.55 (–915.48 to 588.38)	.67	59.37 (–703.91 to 822.64)	.88
	Daily intake of fruits and vegetables^b^	0.25 (–0.39 to 0.89)	.45	–0.04 (–0.56; 0.49)	.89
	Daily intake of high-sodium food^b^	–0.05 (–0.47 to 0.37)	.82	–0.28 (–0.62; 0.06)	.11
	Daily intake of high-fat and high-sugar foods^b^	1.74 (0.65 to 2.83)	.002	0.64 (–0.20; 1.49)	.14

^a^METs: metabolic equivalents.

^b^Mean differences were estimated using linear mixed models using information from the baseline, 6-month, 12-month, and 5-year follow-ups and were controlled by sex and age.

## Discussion

### Main Findings

Although no significant changes were observed in blood pressure levels 4 years after the original intervention was completed, our findings demonstrate important reductions in body weight and BMI. Moreover, participants who received ≥50% of motivational calls during the 1-year intervention potentially benefited most because greater reductions of body weight and BMI were observed; this may have further impact on hypertension incidence, as suggested by the risk estimates. Notably, none of the changes found in the behavioral factors could explain the reductions in body weight and BMI.

### Comparison With Previous Studies

To our knowledge, this is one of the first randomized controlled trials assessing the long-term effects of an mHealth intervention created to promote healthy lifestyle behaviors among subjects at high risk of CVD (ie, with prehypertension) in Latin America. Two relatively recent systematic reviews highlighted the limited number of mHealth interventions in resource-constrained settings, especially from the prevention perspective [6,12].

Among existing studies, Green et al [22] demonstrated that web-delivered pharmacy team care resulted in greater reduction in SBP and improved blood pressure 6-18 months after completion of the interventions. Similarly, Margolis et al [23] showed that intensive intervention based on blood pressure telemonitoring with pharmacist management had sustained effects for up to 24 months (12 months after the intervention ended). Therefore, our study expands on current data by suggesting that mHealth intervention has a sustained effect on body weight 4 years after the intervention ended. On the other hand, Appel et al [24] reported a reduction of 4.6 kg among obese individuals receiving remote support (ie, telephone, website, and email) compared to a control group after 24 months of follow-up; however, no impact on CVD events or all-cause mortality was observed. On the other hand, Rubinstein et al [15] reported no change in blood pressure levels after 12 months of intervention in prehypertensive individuals, but participants in the intervention group had modest reductions in body weight and BMI and reported lower intake of high-fat and high-sugar foods. Recently, a meditation smartphone app appeared to decrease SBP in a 6-month dose-response feasibility trial; however, the adherence to this intervention declined over time [25].

The utility of mobile phone text messages has been reported mainly to support hypertension treatment and management, especially as reminders [26,27]. For example, Bobrow et al [26], using SMS text messages with hypertensive individuals, reported a small reduction in SBP compared to usual care after 12 months of intervention. Similarly, Hacking et al [27] reported that text messages only improved self-reported behavior changes. However, limited literature has assessed the long-term impact of mHealth interventions on the cardiometabolic profiles of prehypertensive individuals.

The GISMAL intervention had a marked long-term impact on body weight and BMI. Previous results of the original study at the Peruvian site showed reductions of 1.24 kg in body weight and 0.53 kg/m^2^ in BMI among those in the intervention arm after 1 year of follow-up (Multimedia Appendix 2) [15]. These new results show that 4 years after the completion of the original study, both body weight and BMI are much lower among individuals who received the intervention than in those who did not. Thus, this 1-year intervention not only helped sustain previous weight loss but also helped ensure a greater weight reduction over time. Surprisingly, despite the clear reductions in weight and BMI at long-term follow-up, there was weak evidence of changes in blood pressure levels. The sample size of the original trial was calculated to detect a difference of 5 mm Hg in each country, and some decreases in SBP and hypertension incidence, especially among those receiving higher doses of the intervention, were noted.

On the other hand, there were no differences among evaluated behavioral factors. These findings suggest that the tools and questionnaires used during the evaluation were not accurate enough to assess selected lifestyles or that the intervention led to changes in unmeasured behaviors that were maintained beyond the period of the study intervention. Other studies have reported similar impacts of mHealth on bodyweight and BMI, but most of them were in the short term [28,29]. Therefore, our results support the fact that a short mHealth intervention comprising motivational interviewing calls and weekly text messages helps participants retain healthy habits and may help them maintain long-term effects.

### Relevance to Public Health

Recently, the American Heart Association and the American College of Cardiology included individuals with prehypertension as having hypertension stage 1 (SBP=130-139 mm Hg or DBP=80-89 mm Hg) and proposed that these individuals need appropriate management [30]. However, although people with prehypertension are at a high risk of developing CVD, they do not receive treatment in resource-constrained settings. This highlights the need for prevention strategies to avoid further complications.

mHealth appears to be a promising way to reduce the risk of these individuals because participants in our intervention group only received monthly health counselling and weekly text messages for 12 months. Regular communication between patients and clinics or health posts may improve adherence to healthy behaviors, which in turn can prevent the onset of CVD later on and contribute to other positive health outcomes [31,32]. In addition, the effects of our intervention could have been greater if booster appointments were utilized, thus extending the behavior changes.

The perceived benefits of this mHealth intervention must outweigh the effort of receiving calls and text messages because self-management is an ongoing process that requires significant iteration. The introduction of apps to support calls and text messages even after the intervention period can help produce sustainable outcomes. However, evidence has demonstrated that in interventions based only on technology, when people are left alone with mobile self-help apps, participants are less adherent [33,34] and less motivated to engage in the proposed program than participants who are accompanied by health staff or coaches or who have other types of face-to-face interaction as part of the intervention [35]. The reductions in mean body weight and BMI indicate possible long-term success of the intervention, with a possible impact on hypertension; hence, this intervention can potentially be implemented to ensure prevention of CVD.

### Limitations

Some limitations must be highlighted. First, this study included only data from participants in Peruvian settings, although the original intervention was conducted in three countries (Argentina, Guatemala, and Peru). However, the effects of the intervention were especially important in Peru, as shown by the 12-month results [15], and the sample size of the trial was calculated separately for each country. Second, the rate of attrition was over 20% after 5 years; thus, some bias may arise in the results. Despite this, the intention-to-treat principle was used in all the analyses. Third, the original intervention was based on the Transtheoretical Model, which is mainly used for smoking cessation [36,37]. Although recent literature shows the use of this model in other interventions [13], the intervention was adapted to be applied for cardiovascular prevention in resource-constrained settings. Fourth, we did not assess differential exposition to other preventive interventions since study randomization. However, as the participants did not have hypertension or any other noncommunicable condition, the effects of this limitation may be negligible. Finally, recall and desirability bias may be present at the moment of evaluation, as is usual in these types of studies. However, validated scales and standardized procedures were used to reduce these biases as in the original study.

### Conclusion

A 1-year mHealth intervention comprising motivational interview calls and text messages appears to have long-term effects on health 4 years after intervention completion. Although we detected no effects on blood pressure levels, important reductions of body weight and BMI were observed. Individuals receiving ≥50% of calls had greater reductions in body weight and BMI, and a potential effect on hypertension incidence was observed. Thus, mHealth appears to be a promising preventive strategy for noncommunicable diseases in resource-constrained settings.

## Appendix

Multimedia Appendix 1 Baseline characteristics of the study population by intervention group.

Multimedia Appendix 2 Outcomes in Peru: comparison after 1-year intervention.

Multimedia Appendix 3 CONSORT e-HEALTH checklist (V. 1. 6. 1).

## Abbreviations

CVD: cardiovascular disease
DBP: diastolic blood pressure
GISMAL: Grupo de Investigación en Salud Móvil en America Latina
mHealth: mobile health
METs: metabolic equivalents
RR: risk ratio
SBP: systolic blood pressure
