# Supplementary material for: Sustainability of mHealth Effects on Cardiometabolic Risk Factors: Five-Year Results of a Randomized Clinical Trial
Source: J Med Internet Res. 2020 Apr 21;22(4):e14595. doi: 10.2196/14595 (PMC7201320; doi:10.2196/14595)
Supplement: Multimedia Appendix 1 [file jmir_v22i4e14595_app1.docx]

**Multimedia Appendix 1:** Baseline characteristics of the study population by intervention group

|  | **Intervention group**  **(n = 107)** | **Control group**  **(n = 105)** |
| --- | --- | --- |
| **Sociodemographic variables** |  |  |
| Age, years |  |  |
| Mean | 44.6 (8.2) | 44.5 (7.8) |
| ≤45 years | 56 (52.3%) | 56 (53.3%) |
| >45 years | 51 (47.7%) | 49 (46.7%) |
| Male sex | 43 (40.2%) | 42 (40.0%) |
| Marital status |  |  |
| Single | 18 (16.8%) | 19 (18.1%) |
| Married or cohabitating partner | 79 (73.8%) | 75 (71.4%) |
| Separated, divorced or widow | 10 (9.4%) | 11 (10.5%) |
| Household income |  |  |
| First quintile | 9 (8.4%) | 8 (7.6%) |
| Fifth quintile | 12 (11.2%) | 12 (11.4%) |
| Years of education | 11.3 (3.6) | 11.3 (3.9) |
| Employment status |  |  |
| Employee | 25 (23.4%) | 24 (22.9%) |
| Independent worker | 49 (45.8%) | 45 (42.9%) |
| Housewife | 32 (29.9%) | 33 (31.4%) |
| Other | 1 (0.9%) | 3 (2.8%) |
| Health insurance coverage | 60 (56.1%) | 70 (66.7%) |
| **Self-reported behavioural variables** |  |  |
| Smoking status |  |  |
| Never smoker | 44 (41.1%) | 41 (39.1%) |
| Former worker | 40 (37.4%) | 42 (40.0%) |
| Current smoker | 23 (21.5%) | 22 (20.9%) |
| Alcohol intake* |  |  |
| Never | 26 (24.3%) | 23 (21.9%) |
| ≤1 time per month | 59 (55.1%) | 61 (58.1%) |
| 2-4 times per month | 18 (16.8%) | 20 (19.1%) |
| 2-3 times per week or more | 4 (3.7%) | 1 (0.9%) |
| Physical activity |  |  |
| METS/min per week | 462 (132 – 796) | 347 (0 – 792) |
| Low physical activity† | 51 (63.0%) | 52 (64.2%) |
| Daily dietary intake (number of servings) |  |  |
| Fruits and vegetables | 1.7 (1.1) | 1.7 (1.4) |
| High sodium foods | 0.8 (0.7) | 0.8 (0.9) |
| High-fat and high-sugar foods | 5.3 (2.4) | 5.2 (2.4) |
| **Stages of change** |  |  |
| Physical activity most days |  |  |
| Pre-contemplation/contemplation | 23 (21.5%) | 27 (25.7%) |
| Preparation/action | 45 (42.1%) | 48 (45.7%) |
| Maintenance | 39 (36.4%) | 30 (28.6%) |
| Intake of 5 serving of fruits and vegetables |  |  |
| Pre-contemplation/contemplation | 35 (32.7%) | 35 (33.6%) |
| Preparation/action | 65 (60.8%) | 63 (60.6%) |
| Maintenance | 7 (6.5%) | 6 (5.8%) |
| Food with harmful fats |  |  |
| Pre-contemplation/contemplation | 8 (7.5%) | 6 (5.7%) |
| Preparation/action | 55 (51.4%) | 52 (49.5%) |
| Maintenance | 44 (41.1%) | 47 (44.8%) |
| High-sugar food and beverages |  |  |
| Pre-contemplation/contemplation | 6 (5.6%) | 5 (4.8%) |
| Preparation/action | 53 (49.5%) | 47 (44.8%) |
| Maintenance | 48 (44.9%) | 53 (50.4%) |
| High-sodium processed food |  |  |
| Pre-contemplation/contemplation | 7 (6.5%) | 5 (4.8%) |
| Preparation/action | 48 (44.9%) | 38 (36.2%) |
| Maintenance | 52 (48.6%) | 62 (59.1%) |
| Salt added at the table |  |  |
| Pre-contemplation/contemplation | 7 (8.6%) | 6 (7.0%) |
| Preparation/action | 5 (6.2%) | 6 (7.0%) |
| Maintenance | 69 (85.2%) | 74 (86.0%) |
| Salt added for cooking |  |  |
| Pre-contemplation/contemplation | 61 (57.0%) | 65 (61.9%) |
| Preparation/action | 46 (430%) | 40 (38.1%) |
| **Physical measurements** |  |  |
| Bodyweight (kg) | 78.7 (13.3) | 79.3 (15.0) |
| Body mass index (kg/m^2^) | 31.5 (4.8) | 32.2 (5.0) |
| Systolic blood pressure (mmHg) | 126.2 (5.7) | 126.6 (6.5) |
| Diastolic blood pressure (mmHg) | 76.6 (6.4) | 77.0 (7.0) |

Data are mean (SD), n (%), or median (IQR). METS = metabolic equivalents of task.

* Alcohol intake and other diet measures were obtained from the Food Frequency Questionnaire.

† Low physical activity refers to less than 600 METS/min/week.
