# Supplementary material for: Sustainability of mHealth Effects on Cardiometabolic Risk Factors: Five-Year Results of a Randomized Clinical Trial
Source: J Med Internet Res. 2020 Apr 21;22(4):e14595. doi: 10.2196/14595 (PMC7201320; doi:10.2196/14595)
Supplement: Multimedia Appendix 2 [file jmir_v22i4e14595_app2.docx]

**Multimedia Appendix 2:** Outcomes in Peru: comparison after 1-year intervention

|  | **Intervention** | | **Control** | | **Difference at 12 months** |  |
| --- | --- | --- | --- | --- | --- | --- |
|  | **Baseline** | **12 months** | **Baseline** | **12 months** |  | **p-value** |
| **PERU** | **N = 107** | **N = 95** | **N = 105** | **N = 98** | **Δ (95%CI)** |  |
| SBP (mmHg) | 126.2 (5.7) | 119.7 (8.7) | 126.6 (6.5) | 120.1 (10.4) | -0.69 (-3.48; 2.09) | 0.62 |
| DBP (mmHg) | 76.6 (6.4) | 72.0 (8.0) | 77.0 (7.0) | 71.8 (8.1) | 0.61 (-1.63; 2.85) | 0.87 |
| Weight (kg) | 78.7 (13.3) | 77.9 (13.1) | 79.3 (15.0) | 79.5 (15.2) | **-1.24 (-2.16; -0.31)** | **0.009** |
| BMI (kg/m^2^) | 31.5 (4.8) | 31.2 (4.7) | 32.2 (5.0) | 32.3 (5.2) | **-0.53 (-0.92; -0.14)** | **0.007** |
